# A New Work on Surgery

**Published:** 1897-09

**Authors:** 


### A New Work on Surgery.

A new work has been issued of which Dr. John S. Marshall is the author. The work has been prepared with special reference to the needs of dentistry, and judging from the reputation and ability of the author, as both a specialist and general surgeon, the work is an invaluable one.

We have not had an opportunity to make a careful examination of the work and hope to have a more extended notice of it in the next number of the REGISTER.

